# Developing a Theory of Community Caring for Public Health Nursing

**DOI:** 10.3390/healthcare11030349

**Published:** 2023-01-25

**Authors:** Saori Iwamoto

**Affiliations:** Department of Public Health Nursing, Kobe City College of Nursing, 3-4 Gakuennishi-machi, Nishi-ku, Kobe 651-2103, Japan; iwamoto@kobe-ccn.ac.jp; Tel.: +81-78-794-8080

**Keywords:** public health nurses, social trust relationship, nursing theory, caring community

## Abstract

Nursing theories focus on individual and community care and human relationships in unique contexts. One of these contexts is the community in which a theory-based systematic nursing practice process is warranted. This article describes a theory of Community Caring for Public Health Nursing (CCPHN), which is grounded in four nursing metaparadigms by Fawcett: persons, environment, health, and nursing. This theory has three assumptions: (1) community caring fosters care demonstrations in nursing, (2) caring communities comprise members with community attachments united by their common values rather than rigid customs, and (3) community caring is expressed competently in mutual-care practices. From these assumptions, a nursing perspective supporting the community caring process is exhibited as the expression of caring by public health nurses toward supportive and promotive nursing processes that enhance a caring community. Nurses play critical roles in leading the establishment of caring communities. In future research, it is critical to verify whether building a caring community by public health nursing practices based on this theory of CCPHN contributes to the health and well-being of the people in the community.

## 1. Introduction

As the aging population increases, so does the demand for community care nursing practices [1]. In Japan, however, community support systems by kinship and resident bonding in villages have declined considerably, resulting in exacerbated isolation among local communities [2]. Owing to the rapidly increasing care needs of older people in home settings, varying family dynamics, and the competency to provide such support, the provision of care among this population continues to decline [3]. The changes in family relationships and traditional cultural values—such as filial piety, which is prevalent in Japanese society—have resulted in challenges such as increased [4] abuse of older adults, consequently leading to reduced support networks. These relationships include poor intra-family networks or neighborhood relationships that become crucial later in life [5]. According to data from Japan’s Ministry of Health, Labour, and Welfare, in 1975, 52.7% of the population was in close contact with their neighbors. In 2004, however, the figure dropped markedly to 22.3% [6]. Moreover, shorter hospital stays lead to patients’ early reintegration into the community [7,8] and have increased the need for the support of public health nurses.

Personal social capital and trust in the community are independently associated with a reduced risk of infant physical abuse [9]. Therefore, it is important to establish social networks and maintain social capital within the community [10]. Mutual support in the community is required both for the early detection of care needs as well as professional support [11,12,13]. 

Public health nursing uses the norms of social justice based on the standards of the activity. Additionally, public health nursing supports the lives of the targeted individuals, families, and communities and builds systems to support the health of individuals, families, and communities through cooperation that targets the related organizations by creating and organizing social resources [14]. 

Public health nurses (PHNs) play an important role in identifying the characteristics of local social capital in the community [15]. Community social capital, including community culture and identifying resources in the community healthcare system, is one of the core functions of PHN [16]. Additionally, community social capital has been linked to favorable health outcomes and life satisfaction [17]. Social inequalities within the population’s social capital present public health challenges. 

In 1986, the World Health Organization (WHO) adopted the Ottawa Charter, which underscores the importance of strengthening community activities to maintain and promote health among individuals. However, widening socioeconomic disparities have led to health disparities [18]. The WHO proposed that social determinants can influence the community as well as the surrounding environment, thus impacting individual health [19,20]. Therefore, nursing care aimed solely at individuals is limited in improving the overall health of community members. Consequently, public health nursing has become increasingly important for developing community structures and social systems.

### 1.1. Valuing the Community Caring Nursing Theory

Theory-based nursing practice demonstrated through community caring is a systematic process of nursing that promotes a safe environment and patients’ health and well-being [21,22,23,24]. Parker and Barry [23] developed the community nursing practice model (CNPM), informed by the premise that the community is a safe place for its members as it ensures the protection and security of all individuals. A community is defined by its members, shared values, and geographical boundaries; the health and well-being of individuals and families within communities are prioritized and nurtured through caring relationships.

According to Robert et al. [25], caring promotes human development and a sense of community, referring to social roles or groups of individuals (such as a nurturing family, a supportive peer group or neighborhood, or a compassionate school) that promote human development and the provision of care. Examples of anti-community groups are destructive dysfunctional families, alienated and hostile neighborhoods, and a school that menaces instead of teaching children. These groups are deemed anti-community as their intentions and actions are not within the caring science perspective. Additionally, its unfortunate outcome is global ill fare and continuing dehumanization [26]. Therefore, it is important to remove these barriers and build a caring community.

Similar to personal care, true community enhances human development by increasing mutual aid and shared heritage among people instead of demeaning, dividing, or imposing values. Furthermore, a true community strengthens families, enables parents and other caregivers to perform their jobs well, and respects individual rights [27]. Thus, in caring communities, nurses provide the best possible care without discrimination or limitations.

However, mutual caring by community members is insufficient because of several challenges exacerbated by the lack of societal structures available to support effective caring. One of these structures is mentioned by Falk-Rafael [28], who asserts that public and community health nursing scholars are dissatisfied with the lack of conceptual frameworks useful in guiding community health nursing practices. Furthermore, Rafael states that Watson’s human caring theory [29] is useful for PHNs to provide care in the community. 

Bent [30] asserted that caring is the key concept in nursing since it relates to other foundational concepts—person, health, and the environment—within the profession and discipline. Crucially, “community caring” and “integrative caring praxis” at the community level remain unclear [30]. A major challenge for nurses is securing an environment to provide engaged and authentic community care owing to the demands and structure of the nursing discipline. To reflect the dynamic praxis of knowing, being, and doing through community health nursing, caring must focus on communities, the environment, and global society [30].

### 1.2. Caring in Nursing Communities and a Community of Caring

Nursing practice must care for the needs of the community, the environment, and global society to be inclusive [30]. Caring requires going around the walls between estranged individuals and between estranged individuals and their communities [31]. PHNs must improve those oppressive structures through civic involvement at every personal and professional level. Additionally, by strengthening individual clients, nurses will enhance empowered communities [31]. For example, nurses in medical facilities can connect patients to care as they seek treatment and visit medical facilities on their own. PHNs, however, identify marginalized persons through outreach activities; PHNs go out into the community themselves or through various networks to identify potential clients in the community. Once PHNs have identified marginalized persons, they will perform community outreach to connect them with people in the community. 

People with mental illnesses are subjected to stigma and discrimination while consistently facing restrictions in the exercise of their political, civil, and social rights [32]. Family members of people with severe mental illness are stigmatized [33]. For those with mental disorders who are marginalized in the community, PHNs work to reduce the stigma of mental illness by helping people in the community understand mental illness. Furthermore, PHNs work to improve oppressive structures against people with mental disorders by creating places in the community where people with mental disorders and their family members can gather. 

Gadow and Schroeder [34] explicated the concept of community as a partner, in which the nurse’s goal is to enhance community self-determination. Moreover, Pope et al. [35] declared that a caring nursing practice works to protect a community’s right to autonomy. In this new partnership model [35], PHNs must develop strategies to meet the healthcare needs of individuals and communities and continue to identify ongoing ethical considerations to ensure a community nursing practice grounded in caring. PHNs must especially recognize the role of policymakers on ethical issues related to healthcare [35]. Specifically, PHNs can use policy methods because they are often responsible for public health in government agencies. The community has the right to care regardless of location, age, sex, ethnicity, medical condition, and economic status [36]. It is important to develop policies that allow marginalized people to have a place in the community, interact with community members, and build a community where no one is inhibited by society. Intentional and informed participation by nursing with policymakers on ethical questions related to healthcare decisions expands the scope of a caring nursing practice and benefits communities and individuals. The community discourse should address issues directly related to patient autonomy, access to healthcare, and resource allocation within the scope of this practice. 

As stated above, caring action is required not only by community PHNs but also by community members. Community caring theory for PHNs is integral to guiding their nursing practice in the community. However, there is no public health nursing theory specifically for PHNs to create a caring community that supports the community and builds relationships between the community members. 

### 1.3. Purpose

The aim of this theoretical paper is to describe the development of the theory of Community Caring for Public Health Nursing (CCPHN). The conceptualization processes addressed include motivation, literature review, assumptions, processes, clinical practice, and educational preparation. This thorough exposition of the development of CCPHN provides the basis and framework for application in practice, education, and research.

## 2. Developing the Theory of CCPHN

### 2.1. Motivation to Develop the Theory

A situation of caring in the community resulting from the 1955 Morinaga arsenic milk poisoning during a time of rapid development in Japan provided the impetus for advancing the development of a formalized theory of community caring in nursing. The situation was an outbreak of poisoning from ingesting arsenic-contaminated Morinaga baby formula, hereafter referred to as “the arsenic milk poisoning incident” [37,38,39]. This incident occurred in western Japan in 1955, where several incidents were reported of infants experiencing strange symptoms such as abdominal distention; blackening of the skin; loss of appetite; debilitation; and, in some severe cases, death. 

Although many infants were treated at medical facilities for these symptoms, the problem was not immediately traced back to the baby formula [40]. A few months after the outbreak of this incident, there was a substantial increase in the number of affected infants. Eventually, it became evident that these symptoms were caused by ingestion of the baby formula manufactured by the Morinaga Milking Industry. The milk was contaminated with arsenic and other heavy metals. The outbreak was one of the most disastrous incidents to affect infants in history, resulting in more than 10,000 victims. After that, a panel of government-appointed physicians surveyed these victims and concluded that it was unlikely that these victims would experience permanent damage.

Morinaga Milking Industry paid temporary compensation to the victims, and for a while, the incident seemed to have been resolved [38,41]. However, 14 years later, PHNs found that many individuals who were victims of the arsenic milk poisoning incident were still experiencing the after-effects of arsenic poisoning but were not receiving due care. The PHNs made house visits to each victim to identify the specific after-effects of arsenic milk poisoning and the suffering experienced by the parents of these victims. Thus, Japan implemented the “14th-year visit”, in which a PHN was required to visit children affected by the Morinaga arsenic milk poisoning incident [37].

The initiative consisted of PHNs visiting each damaged child left unattended, clarifying the actual situation of the damage, and establishing systems and mechanisms to ensure that the children could live safely in the area. PHNs were instrumental in helping children and their families who had been deprived of their rights to compensation and care [40].

The history of the PHN caring response to this disaster motivated the development of the theory of CCPHN to help PHNs identify and care for communities and persons with health conditions and ameliorate or prevent those conditions. Furthermore, PHNs help vulnerable people live in the community while being accepted by its members. PHNs are responsible not only for the health of the individual but also for the health of the overall community. Thus, PHNs targeting such communities should be able to visualize caring for these communities. Caring for the community will lead to community development that includes the health of all members, especially vulnerable groups.

### 2.2. Review of the Literature for Developing the Theory of CCPHN

Establishing relationships between and among theoretical concepts uncovers community healthcare engagements, mutual trust in society, and caring. This informs a PHN’s supportive practice for providing healthcare to local community members.

A search was conducted in January 2022 using PubMed without limiting the year of publication. It was supplemented by a manual search. The search was performed using keywords in English with the following search combination: (“Caring”) AND (“Community”) AND (“Public Health Nursing”). From 1965 to 2022, 864 studies were found. Eleven records were included in the final analysis (Table 1).

The development of this theory included a literature review focused on community healthcare engagements, mutual trust in society, and PHNs’ caring activities for healthcare to local community members. The literature review included studies from several countries and community members with several health conditions. 

The results of the review showed several issues in the community regarding relationships, roles, and responsibilities, which highlight the need for strategies in establishing caring communities. Pindus et al. [42] conducted a systematic review and meta-ethnography among stroke survivors and informal caregivers in primary care and community healthcare facilities. They found caregiver limitations in and interruptions to providing healthcare services for patients owing to issues related to the continuity of care, limited access to services, and inadequate information provision. Stroke survivors and caregivers felt abandoned because they had limited access to services, which was exacerbated by their lack of knowledge or skills to re-engage. This condition demonstrated the lack of support, thus emphasizing the need for greater continuity of care. Ideally, communities and healthcare personnel should provide mutual help to prevent any form of marginalization of community residents with a health condition. To ensure that people with stroke sequelae are not inhibited in the community, it is necessary not only to provide medical care but also to establish support groups such as patient associations and build a place where they can play a role in the community and support others. 

Gilmore et al. [43] conducted a rapid evidence synthesis on community engagement for COVID-19 prevention and control. Their study identified six main groups in community engagement: local leaders, community and faith-based organizations, community groups, health facility committees, individuals, and key stakeholders. All groups serve different functions in the community, including designing and planning, community entry and trust-building, social and behavior change communication, risk communication, patient surveillance and tracing, and logistics and administration. This study suggests that participatory community approaches, such as involving community members and building trust, are also important in COVID-19 prevention and infection control. Supporting communities is a meaningful activity in the prevention of infectious diseases. 

Williams et al. [44] evaluated community partnerships to address community resilience through a social network survey of community coalitions facilitated by PHNs. Their study found that the community resilience coalitions suggested the possibility of approaches by PHNs, such as building partnerships among residents that would increase activities for helping vulnerable groups and promote disaster preparedness resilience communities.

Tsuruta et al. [45] investigated the relationship between social capital and happiness in a community with the lowest national health insurance expenditure in Japan. They found a positive relationship between all three social capital factors (i.e., trust, connections and interaction, and social participation) and happiness among community residents. Both studies showed the need for mutual relationships and help among community residents fostered by PHNs. 

Kawasaki et al. [46] investigated PHNs’ experiences in caring for the community during the recovery from the Fukushima nuclear accident. Results found that despite being powerless owing to incorrect information and insufficient knowledge, PHNs cared for people through their compelling sense of service. This experience was much like what Zerwekh [31] described as fearless caring, based on three meta-themes: the human connection, the community connection, and making self-care possible. Each of these themes is explained as follows:

The human connection: This caring meta-theme is grounded in the nurses’ strongly held conviction that they share a common humanity with disenfranchised people. The human connection is characterized by actions that involve honoring humanity, knowing community members, and sharing one’s humanity [31].

Honoring their humanity: Nurses believe that they are consulted and trusted by their clients because they treat them with respect [31].

Knowing their humanity: Expert nurses practicing with marginalized people can discover what is happening in their clients’ lives [31]. They know their past, are familiar with their patterns, and are experts in asking questions about unearthing clients’ life narratives.

Sharing one’s humanity: Nurses who care for disenfranchised people do not hesitate to share their world with clients and are empathetic toward them [31].

Parker and Barry’s [23,47] CNPM is the synthesis of a decade of experience in caring for persons and communities. The values form the basis of the model as well as nursing practice. The transcendent values of respect, caring, and wholeness align with those of primary healthcare: access, vitality, empowerment, interdisciplinary collaboration, and community participation. Within this model, nurses and social workers are encouraged to reach out by expanding their networks and strengthening their relationships with colleagues, clients, and community members [48]. 

Pope et al. [35] described that it is imperative for the profession to clearly articulate an ethical perspective grounded in responsive relationships with individuals and communities, that is, caring, morals, and values for the practice of all nurses. A challenge to nursing is to engage in a responsive, ethical, and philosophical discourse when the community is whole and autonomous. They explored ethics in community practice with the principles of caring, beneficence, autonomy, advocacy, and social justice. Caring nursing practice must especially work to protect a community’s right to autonomy. Conscious participation in nursing with policymakers on ethical questions related to healthcare decisions expands the scope of a caring nursing practice and benefits communities as well as individuals. The community discourse addressing issues directly related to patient autonomy, access to healthcare, as well as resource allocation is the scope of this practice.

Falk-Rafael’s theory of critical caring [49,50,51] provides the foundation that foregrounds nursing knowledge and values in education. The phrase “critical caring” refers to a relational way of being that enhances and protects human dignity and well-being while simultaneously addressing the social, political, environmental, and economic factors that influence human health. This theory emerged from reflective-theory-guided practice and was further developed through research. Furthermore, it was developed from PHN practice with the intent to articulate the scope and nature of that self-care practice to medical administration and colleagues in other public health disciplines. The theory offered theoretical legitimacy and visibility to PHN practice. Additionally, the theory offered a common language through which the work of PHNs could be described. 

Another finding describes Falk-Rafael’s Critical Caring [28], grounded in nursing through Watson’s caring science [29] and Nightingale’s legacy of social activism and in feminist critical theory. Critical caring has emerged from the experience of expert PHNs. Critical caring is conceptualized as a way of being (ontology), knowing (epistemology), choosing (ethics), and doing (praxis) expressed through seven caritive health-promoting processes derived from Watson’s 10 clinical caritas processes. Additionally, it shows seven health-promoting processes. In particular, the second process, “Developing and maintaining a helping-trusting relationship”, refers to building trust within the community, and the fifth process, “Contributing to the creation of supportive and sustainable physical, social, political, and economic environments”. Moreover, “Meeting the needs and building the capacity of communities and their members” indicates the importance of supporting capacity building that meets the needs of the community. Thus, critical caring [49] is the realization of political and social action from the personal knowledge gained from providing care to the vulnerable. This includes advocacy for social justice and a shift to policy-related reform. 

Falk-Rafael [51] identifies the development of a trusting relationship, advocacy, providing information and developing skills, and capacity building as ways to assist clients. Active participation of the client in their empowerment was essential; they asserted that they could only facilitate, not create, empowerment in others, yet hinted at their responsibility to do so by referring to empowerment as a matter of social justice and equity. In addition to active participation, nurses identified increased awareness as being critical to the empowering process. That awareness was threefold and included awareness of one’s strengths and limitations, one’s rights to have control over personal/family health issues and a voice in decisions directly affecting one’s health, and social and political factors that influence health and healthcare. Interweaving and interacting with increased awareness and participation and increased knowledge and skills made acting on informed choices not only more possible but also more likely to be successful in achieving clients’ desired outcomes and attaining their health goals. Although it began internally, the nurses asserted that empowerment produced “ripple effects” that positively affected family members and others with whom the client interacted. However, the ripples extended not only outward but also back toward the nurse and client. Nurses themselves are empowered through their clients’ empowerment in a reciprocal effect.

Smith et al. [52] developed the Smith-Campbell Caring Community Model. Their case study exemplifies a community that felt a moral obligation and empathy to assist members of their community who had special needs. The caring action led to receptivity and positive outcomes or actual healthcare services for the medically indigent.

**Table 1:** Summary of studies included.

Reference	Research Design	Aim of the Study	Key Findings
Pindus et al. [42]	Systematic review and meta-ethnography	To describe and explain stroke survivors’ and informal caregivers’ experiences of primary care and community healthcare services. To offer potential solutions for how negative experiences could be addressed by healthcare services.	Stroke survivors and caregivers feel abandoned because they have become marginalized by services, and they do not have the knowledge or skills to re-engage. This can be addressed by increasing stroke-specific health literacy through targeted and timely information provision and improving continuity of care between specialist and generalist services.
Gilmore et al. [43]	Rapid evidence review	To identify what approaches and community actors are involved, what interventions are conducted, who the target groups of community engagement are and how equity considerations are incorporated, what the linkages and relationship with other health system stakeholders are, and what the main implementation considerations for successful community engagement for infectious disease prevention and control are.	This rapid review highlights the main community engagement actors and approaches and the interventions that they conduct within the prevention and control of the infectious disease. This review also notes the lack of documented community engagement activities in high-income countries. Well-implemented community engagement strategies can support the design of interventions, building trust, and community entry, social and behavioral change communication, risk communication, surveillance and contract tracing, and logistical and administrative support during COVID-19 prevention and control responses.
Williams et al. [44]	These coalitions were randomized to one of two approaches (community resilience or preparedness)	To clarify a social network survey to measure the number, type, and quality of relationships among organizations participating in 16 coalitions brought together to address community resilience in the Los Angeles Community Disaster Resilience project.	The community resilience coalitions were initially larger and had lower trust among members than the preparedness communities. Over time, these trust differences dissipated. While both coalitions grew, the resilience community coalitions maintained their size difference throughout the project. We also found differences in the types of activities implemented by the resilience communities; these differences were directly related to the training provided.
Tsuruta et al. [45]	A cross-sectional study	To examine the relationship between social capital and happiness in a community with the lowest National Health Insurance expenditures in Japan.	A positive relationship between social capital and happiness concerning all three factors of social capital (trust, connections, interaction, and social participation).
Kawasaki et al. [47]	Qualitative descriptive research	To describe public health nurses’ (PHNs) experiences in caring for people in their communities during the recovery stage of the Fukushima nuclear accident.	The PHNs supported and cared for people in their communities, driven by their compelling sense of mission even in the absence of correct information and sufficient knowledge. They spoke of being heartbroken and barely able to face the reality of the impact of the accident. PHNs supported people because of a compelling sense of mission, yet it was a great burden.
Zerwekh [31]	Phenomenological approach	To learn anew the world of caring, not that previously encoded.	Fear and silencing keep us from “rising up” at all levels of organization and community; the truth is rarely spoken to power for fear of repercussions. As nurses, by strengthening individual clients, we enhance the possibility of them acting as empowered communities. Nurses can validate, explain, teach, and replicate fearless caring with clients subject to innumerable societal injustices and fears. As the gap between rich and poor widens, a unique group of outstanding nurse colleagues persistently struggles to affirm humanity and build the individual capacity of the most disadvantaged. They draw them into communities where there is strength in numbers. They see human possibilities where others see no hope. Thus, power is born when caring for others, valuing another, and believing in human potential. Experiencing concern and unconditional regard, the patient’s self-love and self-regard gradually increase.
Parker and Barry’s [23,47]	Theoretical paper	To describe the application of a community nursing practice model to school nursing.	The first concentric circle includes persons and groups in each school or community, such as parent/teacher organizations, who share their concern for the well-being of the persons being served. The categories of care that comprise the first circle are consultation and collaboration and appraisal and evaluation. The second circle includes structured and organized groups within a wider range or jurisdiction, such as a district- or county-level organization, which also share a concern for the well-being of persons being served. The categories of care include consultation and collaboration as research and evaluation. The third circle reaches out to state, regional, national, and international organization members from whom consultation, collaboration, appraisal, and evaluation are sought.
Pope et al. [33]	Theoretical paper	To explore ethics in community practice with the principles of caring, beneficence, autonomy, advocacy, and social justice.	The ethical principles of beneficence, autonomy, advocacy, and social justice in community nursing were discussed from the lens of caring. Caring in nursing practice must work to protect a community’s right to autonomy. Conscious participation by nursing policymakers on ethical questions related to healthcare decisions expands the scope of a caring nursing practice and benefits communities as well as individuals. The community discourse addressing issues directly related to patient autonomy, access to healthcare, and resource allocation is within the scope of this practice.
Falk-Rafael [49]	Theoretical paper	To examine and congruence of critical caring theory with public health nursing practice.	Congruence between expert public health nursing practice and the theory in terms of caring/social justice ethics that underpins practice and the relevance to their practice of the carative health-promoting process of contributing to the creation of supportive and sustainable physical, social, political, and economic environments. PHNs encountered many barriers to a practice underpinned by a caring/social justice ethic, some limiting their moral agency.
Chinn, P.L.; Falk-Rafael, A. [50]	Theoretical paper	To present a theoretical model that grounds teaching and learning in nursing on the focus, values, and ideals of nursing as a discipline.	The critical caring pedagogy model was formed by integrating Falk-Rafael’s theory of critical caring in public health nursing, Noddings’ philosophy of caring education, and Chinn’s theory of peace and power. This model of critical caring pedagogy was developed by a logical analysis of the three organizing constructs and the conceptual relationships between and among these constructs. When nurse educators ground teaching and learning practice in nursing’s own theoretical and philosophic foundation, they teach nursing in powerful ways that show nursing values and ideals through action, revealing deeper meanings of the words that form texts, lectures, and objectives set forth in a curriculum outline.
Falk-Rafael, A. [51]	Qualitative exploratory study	To identify their conceptualization of empowerment, the strategies they identified as empowering, and the outcomes of empowering strategies they observed in their practice.	Active participation of the client in their empowerment was essential; they asserted that they could only facilitate, not create, empowerment in others, yet they hinted at their responsibility to do so by referring to empowerment as a matter of social justice and equity. In addition to active participation, nurses identified increased awareness as being critical to the empowering process. That awareness was threefold and included awareness of one’s strengths and limitations, one’s rights to have control over personal/family health issues and a voice in decisions directly affecting their health, and social and political factors that influence health and healthcare. Interwoven and interacting with increased awareness and active participation, and increased knowledge and skills that made acting on informed choices not only more possible but also more likely to be successful in achieving clients’ desired outcomes and attaining their health goals.
Smith-Campbell B. [52]	Case study	To describe the concepts and relationships within caring, the Smith-Campbell Community Model was developed from a community case study.	The case study exemplifies a community that felt a moral obligation and empathy to assist members of their community who had special needs. After interacting with each other and gaining more information about those in need, this plurality of persons felt compelled to act by establishing an organization to meet the needs of the medically indigent. The caring action led to receptivity and positive outcomes or actual healthcare services for the medically indigent. This case study explicated the concepts of caring previously identified in the literature but was broadened to include an ontology of community and individuals.

### 2.3. Concepts Relevant to Structuring Community Caring Practice 

From the aforementioned studies, key points were derived from developing the structure of the theory. Concepts relevant to structuring community caring practices for PHNs are presented and discussed. Namely, (1) a community is an independent entity of people; (2) people in the community have mutual roles; (3) communities and PHNs have mutual responsibilities; (4) PHNs aim to foster a caring community by facilitating the empowerment of the community and enhancing confidence in the local population; (5) the community does not exclude vulnerable people, it protects them and facilitates their empowerment; (6) caring for the community also includes policymaking. As a general policymaking process, PHNs, in collaboration with community residents and related organizations, clarify the individual needs of the community, explain that it is a problem for individuals as well as for the entire community, obtain an administrative budget based on the identified problem, and form policy. This includes the collaboration of community residents and related organizations. These six elements provide the structure from which the theory of community caring has evolved.

#### 2.3.1. Assumptions of the Theory of CCPHN

**Caring is a concept central to professional nursing practice and discipline** [53]

The goal of nursing has remained unchanged; that is, to provide a safe and caring environment that promotes human health and well-being [54]. PHNs working in the community will gain their trust by cooperating with them and creating situations where people feel mutual respect. It is vital to work to produce caring environments for socially vulnerable people in the community. 

**Encounters of caring and being cared for illustrate a common pattern of caring expressions** [55]

The goal of CCPHN is to establish relationships of caring to enhance the health of community residents and protect socially vulnerable people. Occasions such as these are encounters of caring that are mutually inclusive experiences between PHNs and community residents. Understanding living caring values and knowing oneself is the basis for knowing the other as a caring person. 

**Caring in nursing is a mutually lived experience between the nurse and the person being nursed** [56]

Boykin and Schoenhofer (1993) proposed [56] that when communicated intentionally and with authentic presence and interconnectedness, caring is expressed as a sense of oneness with self and others. They declared that nursing is embedded within the nursing situation, defined as a shared lived experience in which the caring between the nurse and community members enhances personhood. The thoughtful reflection upon practical nursing situations provides opportunities for uncovering the knowledge and essence of caring in nursing.

#### 2.3.2. The Process of Nursing in the Theory of CCPHN [Figure 1]

The three nursing processes reflect engagement in the encounter of caring between the nurse and individuals, their families, and the community. These processes are:

1. PHNs regard the community as an independent entity, aim to build a caring community, and facilitate the empowerment of community members.
2. The caring community fostered by PHN creates a sense of solidarity from new values without being bound by old customs that are limiting. PHNs are partners with the community.
3. Forming a caring community involves policymaking. To build a caring community that includes marginalized people, PHNs need to influence the entire community and create measures that meet regional challenges, including the development of new social resources. This will lead to an environment of enhanced health potential.

**Figure 1:**
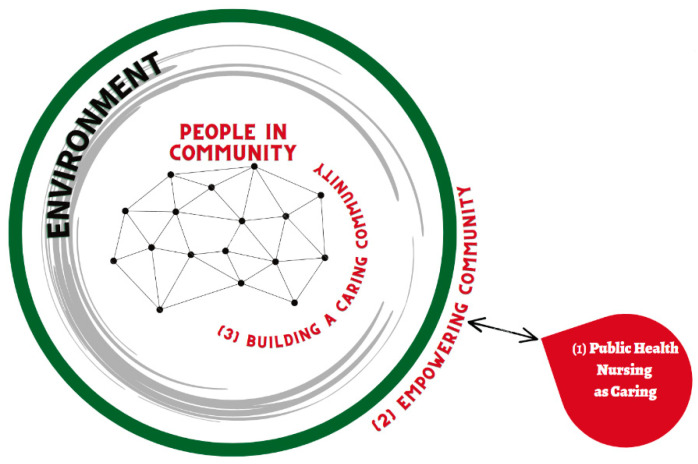
Three nursing processes in the theory of community caring for public health nurses.

#### 2.3.3. Metaparadigm Concepts of Nursing in Relation to the Theory of CCPHN

The metaparadigm concepts of nursing [57,58,59] inform the structure of the theory. The concepts of nursing, persons, environment and health organize the ontological and epistemological descriptions expressly elucidated in community nursing practice. PHNs support the community and will help establish a caring community where members hold mutual values, help each other, and embrace vulnerable people. In such a community, members are the elements that comprise the community, and a caring relationship is present. Structured along the four metaparadigm concepts of nursing: persons, environment, health, and nursing, the theory evolved from a historical health-related incident involving infant milk and societal responsibilities. 

**Nursing:** PHN targets every individual in the community and simultaneously the community itself. PHN activities include not only directly supporting individuals but also creating new policies, projects, and structures to build healthy communities. In PHN, through such activities, the goal is to build an environment in which no one is excluded from the community, and each can live a healthy and happy life.

**Environment:** The environment surrounds and is the community itself. People’s health is affected by their surrounding environment. For people, the community is also one of the environments. In the community, people’s relationships are flexible and fluctuate to establish diverse relationships depending on the time and region. The environment comprises various things such as nature, history, transportation, and policies. The environment can affect the health of individuals and communities. PHNs aid community members in establishing relationships within a caring milieu, invoking and creating social trust relationships (social capital) among community residents. PHNs should develop measures to achieve this.

**Health:** Health is a characteristic of people and the community. Healthy people indicate that people in the community are in good health. As well as individual health, there is community health. Community health implies that every member of the community is healthy and can contribute to individual health through mutual support among members. A healthy community is one that does not exclude the socially vulnerable and in which each individual helps one another. The health of a community affects the health of its members. A caring community is a form of a healthy community.

**Person:** In this theory, the person is an individual, family, or group living in their community. Persons make up a community, each having a mutual relationship and supporting the other. Additionally, each individual and family have their own lived experiences and stories. However, people in the community have various problems, and community caring allows them to demonstrate caring. 

## 3. Community Caring in Nursing Transforming Communities

### 3.1. Caring Competencies and Outcomes of PHN

The concept of “PHN as caring” is important for the community’s health. PHNs strive to create a caring community where people build caring relationships with each other, resulting in improved health outcomes for all members, with no social exclusions. To build a caring community, PHNs use competencies for community care. This competency provides a community structure that allows PHNs to meet and support individuals who have been socially excluded or are vulnerable while simultaneously allowing them to live healthy life without being excluded. Thus, caring is a competency that should be developed.

#### 3.1.1. Public Health Nursing as Caring

Caring associated with PHNs includes helping to improve the attitude, mindset, and sense of responsibility of the community. Caring for the community includes valuing and allocating PHNs for the community and promoting health among individuals. This creates a sense of responsibility for the PHN in charge.

#### 3.1.2. Competency of PHN to Develop a Caring Community

The competency of PHNs to develop a caring community refers to establishing a community where all people are equally valued members, each contributing according to their circumstances. 

**Figure 2:**
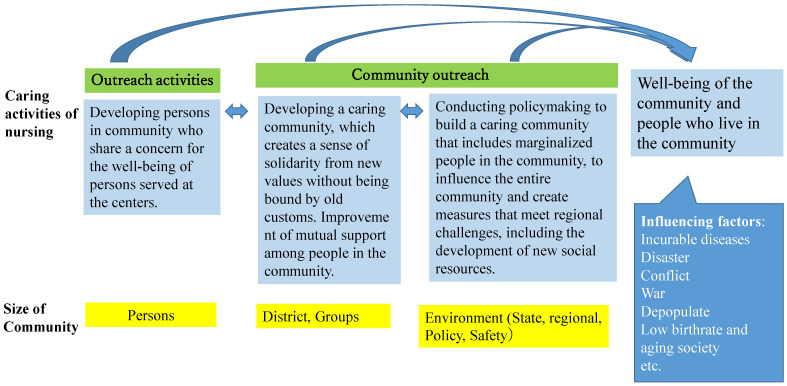
The model of the caring activities of nursing in the theory of community caring for public health nurses.

In the model, the mutual arrow between PHNs and the community indicates that the PHN and community mutually support each other. The motivation for PHNs to establish a caring community is an awareness of strengths and vulnerabilities within the community and the capacity to improve the health of the community. PHNs work with the community to establish a community that includes socially vulnerable individuals, such as those with mental illnesses and isolated mothers.

i. Community Caring Targets and Activities [Figure 2]

The health professional’s target audience for caring is individuals (persons), communities (districts and groups), and environments (the state, region, policy, and safety). PHNs reach out to socially vulnerable individuals who need support but are not receiving it. Additionally, they connect and facilitate the empowerment of these individuals with the necessary support. PHNs reach out to the communities where these individuals live to help create a place for them in the community and build relationships that will enable them mutually support each other. Furthermore, building systems and policies are required to ensure that socially vulnerable people are not marginalized.

#### 3.1.3. The Expected Outcomes for a Caring Community

The expected outcomes refer to building on the strengths demonstrated in traditional customs, helping the community incorporate new values as they desire. It is a state of community where members have symbiosis, mutual aid, and compassion, and there is no social exclusion. Consequently, PHNs will contribute to the health and well-being of the community and its inhabitants.

#### 3.1.4. Factors Affecting Caring Communities

Factors affecting a community’s ability to care to include population aging, declining birth rates, economic disparity, war, disaster, depopulation, and lack of healthcare resources. This can have a negative impact or turn into a positive one. Nevertheless, it is important for PHNs to change negative factors into positive ones by identifying community health challenges and strengths and taking necessary policy steps to address them.

## 4. Clinical Applications of the Theory of Community Caring in Public Health Nursing

### 4.1. Use of this Theory in Practice in the Community

#### 4.1.1. Community Use

PHNs lead and support the health of the community. PHNs care for the community as well as for individuals.

This theory provides the basis for PHNs to support the community. As PHNs promote the health and well-being of people in the community, establishing a caring community is vital. The caring community is a foundational element in building this theory and refers to the relationship in which the community members mutually help each other. 

Studies on social capital [60] have shown that areas with stronger social capital exhibit improved health outcomes. Therefore, establishing a caring community is expected to impact every member’s health positively. 

In addition, PHNs support vulnerable people in the community. It is critical to include vulnerable people when establishing a caring community. Furthermore, it is pertinent to establish a community where vulnerable people can live comfortably. For example, PHNs felt that people with mental illness were isolated from the rest of the community and began promoting mental illness awareness. Moreover, the PHNs highlighted the need to create a place for all people in the community to gather and interact instead of isolated gatherings of people with mental illnesses. Interactions of this nature can help community members establish relationships where they can respond and cooperate to integrate with vulnerable people and establish a community without an isolated person or family.

Healthcare is crucial to fostering a healthy and happy community, an attachment to the community, and a sense of responsibility. PHN nurses and all nurses should study caring science to prepare themselves to nurture caring communities. Deep knowledge of caring science will help eliminate the inclination toward the nurse as a giver and the community member as a receiver and promote the sense of nurse and community members being in partnership to advance the wellness of the community as a caring one. Moreover, PHNs’ skills in establishing a caring community will help develop one with healthy and happy members. Thus, the theory of community caring can be used to improve the health of community members.

#### 4.1.2. Use of this Theory in Basic Education for PHNs and in-Service Education

PHNs should recognize the requirements for providing care in the community. Boykin and Schoenhofer [55] state that persons are caring by virtue of their humanity and further note that while caring is innate, caring competency must be intentionally developed through study and reflective practice. Similarly, all PHNs who care for the community genuinely care about the community and its members. This may result from an attachment to the community or a desire for the survival and development of the community. This type of caring intent is characterized by a strong motivation to improve support for the community.

PHNs can use this caring model when addressing vulnerable individuals. This theory aims to establish a community where these individuals can spend a healthy and happy time in a caring community. Caring for vulnerable people by PHNs is caring for individuals; however, it extends to community caring that includes all people in the community, not just caring for a specific individual. PHNs need to empower their caring competency with the in-depth and continuing study of caring science. Furthermore, clarifying and mastering this nursing art will facilitate the goal of establishing a caring community. This theory can be applied practically to a caring community supported by PHNs educated in caring science.

### 4.2. Future Research

Initial conceptualization of the CCPHN is a meaningful first step in theory-building. With this foundation in place, it is critical to verify whether building a caring community by PHN practice based on this theory of CCPHN contributes to the health and well-being of the people in the community. Moreover, it is necessary to verify whether people’s health and happiness improve when the caring relationship between the community members is strong. Further research to test it with diverse client populations and larger sample sizes would contribute significantly to the sciences of both nursing and public health.

## 5. Conclusions

As expressed by PHNs’ community caring competencies for supporting communities, PHNs who provide competent care facilitate improved community caring in nursing. PHN should establish caring relationships that contribute to people’s health and foster a social relationship based on trust (social capital). Furthermore, PHNs must contribute toward enhancing the vitality and health of local community members and protecting the socially vulnerable. It is critical for researchers to verify whether building a caring community by public health nursing practice based on this theory of CCPHN contributes to the health and well-being of the people in the community.

## Data Availability

Not applicable.
